# Towards optical polarization control of laser-driven proton acceleration in foils undergoing relativistic transparency

**DOI:** 10.1038/ncomms12891

**Published:** 2016-09-14

**Authors:** Bruno Gonzalez-Izquierdo, Martin King, Ross J. Gray, Robbie Wilson, Rachel J. Dance, Haydn Powell, David A. Maclellan, John McCreadie, Nicholas M. H. Butler, Steve Hawkes, James S. Green, Chris D. Murphy, Luca C. Stockhausen, David C. Carroll, Nicola Booth, Graeme G. Scott, Marco Borghesi, David Neely, Paul McKenna

**Affiliations:** 1 SUPA Department of Physics, University of Strathclyde, Glasgow G4 0NG, UK; 2 Central Laser Facility, STFC Rutherford Appleton Laboratory, Oxfordshire OX11 0QX, UK; 3 Department of Physics, University of York, Heslington, York YO10 5DD, UK; 4 Centro de Láseres Pulsados (CLPU), M5 Parque Científico, 37185 Salamanca, Spain; 5 Centre for Plasma Physics, Queens University Belfast, Belfast BT7 1NN, UK

## Abstract

Control of the collective response of plasma particles to intense laser light is intrinsic to relativistic optics, the development of compact laser-driven particle and radiation sources, as well as investigations of some laboratory astrophysics phenomena. We recently demonstrated that a relativistic plasma aperture produced in an ultra-thin foil at the focus of intense laser radiation can induce diffraction, enabling polarization-based control of the collective motion of plasma electrons. Here we show that under these conditions the electron dynamics are mapped into the beam of protons accelerated via strong charge-separation-induced electrostatic fields. It is demonstrated experimentally and numerically via 3D particle-in-cell simulations that the degree of ellipticity of the laser polarization strongly influences the spatial-intensity distribution of the beam of multi-MeV protons. The influence on both sheath-accelerated and radiation pressure-accelerated protons is investigated. This approach opens up a potential new route to control laser-driven ion sources.

## Abstract (web summary)

Intense laser pulse interaction with ultra-thin foils constitutes a promising approach for proton acceleration. Here the authors show that the degree of ellipticity in the laser beam polarization can be used to control the proton beam profile.

## Graphical abstract

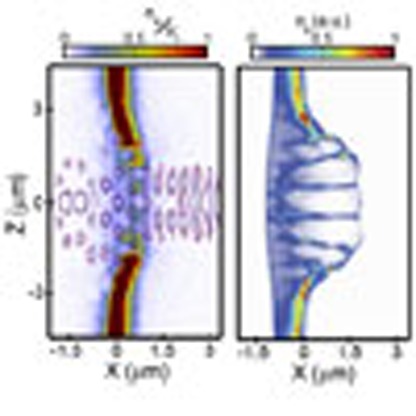

The use of high-power (multi-terawatt to petawatt) laser pulses to drive collective electron motion in plasma has given rise to compact laser-based particle accelerators with potentially wide-ranging applications in science, industry and medicine^1^,^2^,^3^. Control over the collective motion of high-energy plasma electrons enables the resulting beam properties to be varied and is therefore fundamental to the development of these promising sources. A pertinent example is the laser-wakefield acceleration of electrons in low-density plasma by the formation and control of ionized channels^4^ or ‘bubbles'^5^ created by electron expulsion and re-injection. The introduction of increasingly sophisticated techniques to control the bubble evolution and the electron dynamics within it have had a transformational effect on the electron beam energies, energy spread, current and beam stability achieved^6^. It has also led to new types of secondary radiation sources, such as betatron^7^,^8^, and new applications such as phase contrast imaging^9^. By contrast, control of charged particle motion in plasma, which is too dense for laser light to propagate (termed overdense) is significantly more difficult to achieve. In such plasma, the laser light penetrates only to the region of the critical density (where the plasma frequency is equal to the laser frequency), at which point the relativistic electrons produced escape the influence of the laser field. Yet, intense laser pulse interactions with overdense plasma, and in particular thin solid density foils, have, for more than a decade, been shown to be important for ion acceleration^2^,^3^, high flux bremsstralung production^10^, high harmonic generation^11^,^12^, terahertz emission^13^,^14^ and potentially nonlinear, high-energy synchrotron emission^7^,^15^. Controlling the collective motion of high-energy plasma electrons in thin foils would enable new perspectives for developing and applying these unique particle and radiation sources.

The case of an ultra-thin foil, which becomes transparent to the laser pulse during the interaction, is particularly interesting. This can occur via laser radiation pressure-driven compression of the target electron layer to a thickness less than the corrected skin depth for laser penetration. It can also be induced by the relativistic increase in the mass of the plasma electrons oscillating in the laser field, which decreases the frequency of electron oscillations in the expanding plasma to below the frequency of the laser light; a process termed relativistic- (or self-) induced transparency (RIT)^16^,^17^,^18^,^19^,^20^. Laser-overdense-plasma interaction phenomena are induced during the rising edge of the laser pulse, and after RIT occurs the remainder of the pulse is transmitted, driving interaction and collective electron motion within the target volume. The onset of transparency curbs the promising radiation pressure acceleration (RPA) mechanism^21^, but also results in transparency-enhanced ion acceleration schemes such as breakout afterburner^22^,^23^.

It has also been shown to result in the formation of an electron jet, leading to energy enhancement in sheath-accelerated protons^24^,^25^ and to ion beams with a relatively narrow energy spread^26^. Understanding the collective response of plasma electrons to transparency and how this affects the acceleration of ions is important to the interpretation of experiments on ion acceleration from ultra-thin foils. Control of this collective electron motion and the resultant electrostatic fields could enable unprecedented control over laser-driven ion acceleration.

In an important step towards realizing this overall objective, we recently demonstrated that the onset of RIT in an ultra-thin foil in the region of the most intense part of the focused laser pulse can produce a ‘relativistic plasma aperture', resulting in diffraction of the transmitted intense laser light^27^. The controllable structures generated in the near-field diffraction pattern induce transverse ponderomotive forces to which the plasma electrons collectively respond. It was shown that structure in the beam of accelerated electrons can be made to rotate and at an angular rotational frequency that can be controlled by variation of the ellipticity of the laser beam polarization.

Here we demonstrate, experimentally and numerically, that the spatial-intensity distribution of the beam of laser-accelerated protons in ultra-thin foils undergoing transparency is strongly affected by the collective electron dynamics induced by the near-field diffraction pattern. We show that the degree of ellipticity in the laser beam polarization strongly influences the proton beam profile and can thereby potentially be used to control it. The protons are sourced in hydrocarbon layers on the front and rear surfaces of the target foil. The influence of the collective electron dynamics on the protons originating in each layer is investigated. The results highlight a potential new route to controlling the spatial-intensity distribution of these promising ion beams.

## Results

### Role of laser polarization

**Figure 1:**
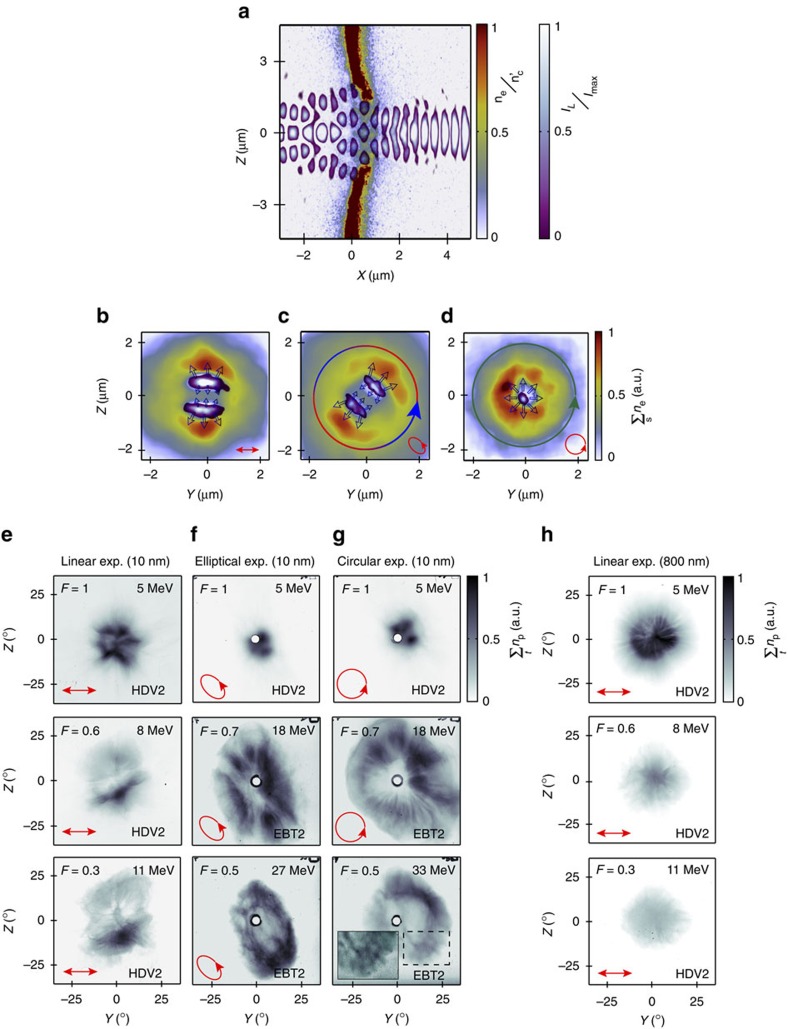
Proton beam experiment results and simulations illustrating laser diffraction through a relativistic plasma aperture (**a**) Example 3D-PIC simulation results showing plasma electron density (*n*_e_) and laser intensity (*I*_L_) in the *X*–*Z* plane at *Y*=0, at a fixed time after transparency has been induced in a 10 nm Al foil. The laser is linearly polarized along the *Y* axis, and the near-field diffraction pattern has a double lobe distribution along the *Z* axis in the region of maximum electron density (*X*∼1 μm). (**b**–**d**) The plasma electron density and laser diffraction pattern in the *Y*–*Z* plane, with the electron density integrated over a laser wavelength, *X*=0.7−1.5 μm, for (**b**) linear, (**c**) elliptical and (**d**) circularly polarized laser light. The small red arrows denote the polarization and the hollow black arrows illustrate the direction of the ponderomotive force arising from the gradients in laser intensity. Plots (**a**–**d**) are discussed in detail in ref. 27. (**e**–**g**) Representative experiment results showing proton beam density distributions at stated energies, as measured at *X*=3.4 cm and with a 10 nm-thick Al foil target, for (**e**) linear, (**f**) elliptical and (**g**) circularly polarized laser light. The colormaps are scaled by the stated value *F* to enable the features of interest at each energy slice to be clearly seen. The inset in (**g**) is further scaled to show the bubble-like density modulations. (**h**) An example reference result for a 800 nm-thick Al target foil, which does not undergo transparency.

We investigate the influence of laser polarization on the spatial-intensity distribution of the proton beam, as a function of proton energy, using high-contrast laser pulses and 10 nm-thick aluminium target foils (see Methods section). Our previous investigation of collective electron dynamics in foils undergoing RIT has shown that high contrast pulses and ultra-thin targets are essential for generating the conditions for which a relativistic plasma aperture, with a diameter of a few times the laser wavelength, is induced^27^. In this case, as shown by the example three-dimensional 3D particle-in-cell (PIC) simulation result in Fig. 1a (from ref. 27), the near-field diffraction pattern of the intense laser light propagating through the aperture forms a double lobe profile in the plane perpendicular to the propagation axis, at the position along the axis at which the target density is highest—the target having already been deformed (pushed forward) by laser radiation pressure on the rising edge of the pulse. As demonstrated in ref. 27, in the case of linear polarization the double lobe profile is fixed in space and orientated perpendicular to the laser polarization axis (the *Y* axis), as shown in Fig. 1b. Electrons respond to the resulting transverse ponderomotive forces and are predominantly pushed outwards to form two density lobes, one either side of the polarization axis. In the case of circular polarization the phase difference gives rise to a laser field vector, which rotates about the propagation axis with a constant angular velocity, once per laser period (Fig. 1d). Electrons are expelled to form a ring-like density profile due to the radial ponderomotive force. For elliptical polarization the angular velocity of rotation of the double lobe and the magnitude of the laser electric field both vary over the laser period. Electrons respond to this angular variation in the radial ponderomotive force to produce density lobes oriented perpendicular to the mean axis of polarization (Fig. 1c). The physics underpinning changes to the laser transmission pattern and the resulting collective electron dynamics is discussed in detail in ref. 27.

Figure 1e–g shows measurements of the spatial-intensity distribution of the beam of protons, at given example proton energies, for linear, elliptical and circular polarization, respectively. These measurements were made during an experiment using the Gemini laser at the Rutherford Appleton Laboratory, UK, as discussed in the Methods section. In all three polarization cases, the target was 10 nm-thick Al and was irradiated by a 40 fs duration (full-width at half-maximum; FWHM) pulse with energy equal to (2.0±0.2) J, focused to a spot size of ∼3 μm (FWHM), producing a calculated intensity equal to 6 × 10^20^ W cm^−2^. These results clearly demonstrate that the proton beam profile is strongly affected by the laser polarization and that the profiles change with proton energy. Additional measured proton beam profiles and measurements of the transmitted laser light (in the far field) are provided in the Supplementary Information File.

The low energy (∼5 MeV) distribution in the linear case exhibits a modulated annular distribution, with radial spoke features and higher densities along the *Z* axis, at *Z*=±7°, that is, either side of the laser polarization axis. At higher energies (∼8 and ∼11 MeV) a larger and lower density distribution is measured, exhibiting a double stripe or lobe pattern. In the elliptical and circular polarization cases, the ring profiles produced at low energy are very small and strongly modulated, whereas at higher energies (∼18 MeV) much larger annular density profiles with clear radial modulations are produced. The ring profiles are circular and elliptical for the respective polarizations and in the latter case the major axis is aligned at an angle similar to the major axis of polarization in the *Y*–*Z* plane. The ring size decreases in both polarization cases for higher energies (for example, ∼27–33 MeV, as shown in Fig. 1f,g). For circular polarization the radial profile is replaced by bubble-like modulations, similar to those reported in ref. 28 and attributed to a Rayleigh–Taylor-like transverse instability^28^,^29^. Note that the upper proton energy detection threshold was significantly higher for the circular and elliptical polarization cases due to the use of a higher-sensitivity dosimetry film in the high-energy part of the detector stack. Finally, to verify that the observed structures are produced only when transparency occurs, a representative measurement with a 800 nm-thick Al target, which does not become relativistically transparent for these laser pulse parameters, is shown in Fig. 1h. The laser pulse is linearly polarized with the same parameters as for Fig. 1e. The proton beam produced with the thicker target does not exhibit the same structures and the divergence decreases with increasing energy, as is typical for proton beams produced by the target normal sheath acceleration (TNSA) mechanism^30^. The Supplementary Information contains further measurements of the structure in the proton beam as a function of target thickness.

To determine how the collective electron dynamics during RIT influences proton acceleration and specifically the role of polarization in defining the proton beam profiles, 3D PIC simulations have been performed in which correlations between the laser, electron and proton distributions are investigated. In a first set of simulations, hydrocarbon layers are included on both surfaces of the Al target foil, which is representative of the target foil conditions in the experiment (protons are sourced in the hydrogen-containing layers, which build up on the target surfaces at the vacuum chamber pressures typically used). To investigate the origin of the two measured distinctive proton populations, we have also separately tracked the protons accelerated at the target front surface, as driven forward by laser radiation pressure (that is, RPA), and the rear surface, as prominently produced by TNSA. Both of these mechanisms are effective before the target becomes transparent.

**Figure 2:**
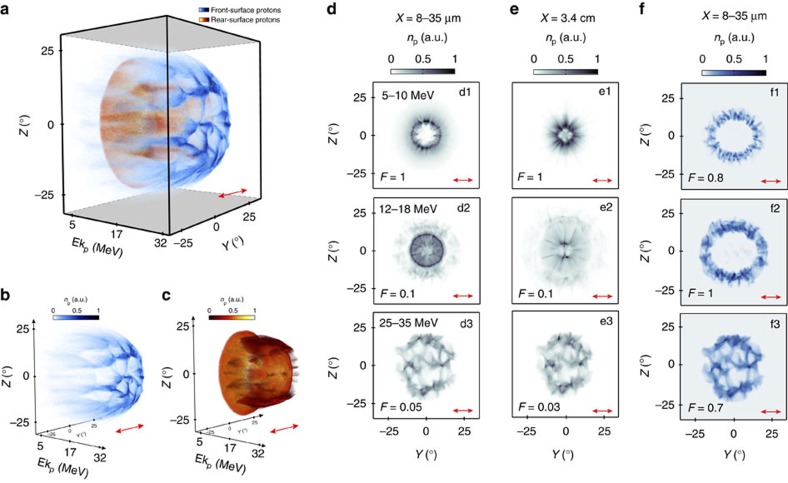
3D-PIC simulation results showing the proton spatial-density distribution for linear polarization. (**a**) Energy-resolved two-dimensional proton density distribution at *X*=8.5 μm. The blue and red distributions correspond to protons originating at the target front and rear surfaces, respectively. The same two distributions are plotted separately in (**b**) and (**c**) for clarity. (**d**) Combined (front and rear sourced) proton spatial-density distributions over the range *X*=8–35 μm, integrated over the stated energy ranges. (**e**) Projection of the combined proton density to *X*=3.4 cm (for comparison with experiment). (**f**) Proton distribution over the range *X*=8–35 μm arising only from protons sourced at the front side of the target foil. The colormaps are scaled by the stated value *F* to enable the features of interest at each energy slice to be clearly observed. The laser is polarized along the *Y* axis and the results in (**d**–**f**) are sampled at *t*=380 fs.

We consider first the linear polarization result shown in Fig. 2. The spatial-intensity profiles of the proton populations sourced at the front and rear sides of the target are distinctly different, as observed when comparing the combined plot in Fig. 2a and the separate plots in Fig. 2b,c. Figure 2d shows the integrated beam profile in the *Y*–*Z* plane (comprising protons sourced in both layers) at three example energy ranges as extracted from the simulation box (integrated over *X*=8–35 μm). Figure 2e shows a projection of the same beam profiles to *X*=3.4 cm (that is, the position at which the experimental measurements are made), as calculated using the proton momentum components in [*X*,*Y*,*Z*] extracted from the simulation results.

At low energies, two distinct features in the proton beam are observed; a high-density, ring-like proton distribution with radial (spoke) features and a spatially larger, lower density halo feature. The ring has a radius of ∼9° at *X*=8–35 μm, decreasing to ∼6° at *X*=3.4 cm, indicating a small reduction in divergence. The density is higher at the top and bottom parts of the ring (that is, along the *Z* axis). The shape, size and radial distribution are all in good agreement with the experiment results shown in Fig. 1e. In the simulation result projected to *X*=3.4 cm at the higher-energy range 12–18 MeV (Fig. 2e) the ring collapses to form a double lobe distribution in *Z*, which has similar characteristics (spatially larger, lower density and double-lobed) to the experiment results at ∼11 MeV in Fig. 1e.

We note that in the experiment results the proton density is asymmetrically distributed across the double lobes and that the lobes are at an angle to the polarization axis, whereas in the simulation results the lobes are more symmetrical and not rotated. This difference is fully accounted for by the fact that the laser focal spot in the experiment is slightly elliptical and asymmetric, with the major axis at an angle to the polarization axis, whereas in the simulations an idealized, symmetric Gaussian focal spot is used. A detailed discussion, with additional simulation results obtained with a focal spot matching that of the experiment, is provided in the Supplementary Information File. A very close agreement between experiment and simulation is found when this correction factor is included. As this effect is secondary to the overall beam profile as defined by the near-field diffraction pattern of the transmitted laser, and can be corrected for in future experiments (for example, by the use of higher resolution adaptive optics), the more general case of a Gaussian focus is used in the simulations throughout the paper.

At even higher energies the beam is dominated by a distribution with modulated stripes. An experimental measurement at this high energy was not achieved in the linear polarization case due to the low sensitivity of the dosimetry film type (HDV2) used. Figure 2f shows only the protons sourced at the target front surface and is directly comparable with the integrated (front and rear) results in Fig. 2d. It is clear that the high-energy striped distribution and low-density halo feature at low energy are produced by protons accelerated at the target front surface. The fact that there are two separate proton components explains the apparent increase in the proton beam divergence with increasing proton energy, observed in the integrated proton distributions in both the experiment and simulation results. The component with higher divergence is produced by front-surface protons, which are accelerated by radiation pressure as the target is undergoing deformation. This is discussed in more detail below.

**Figure 3 f3:**
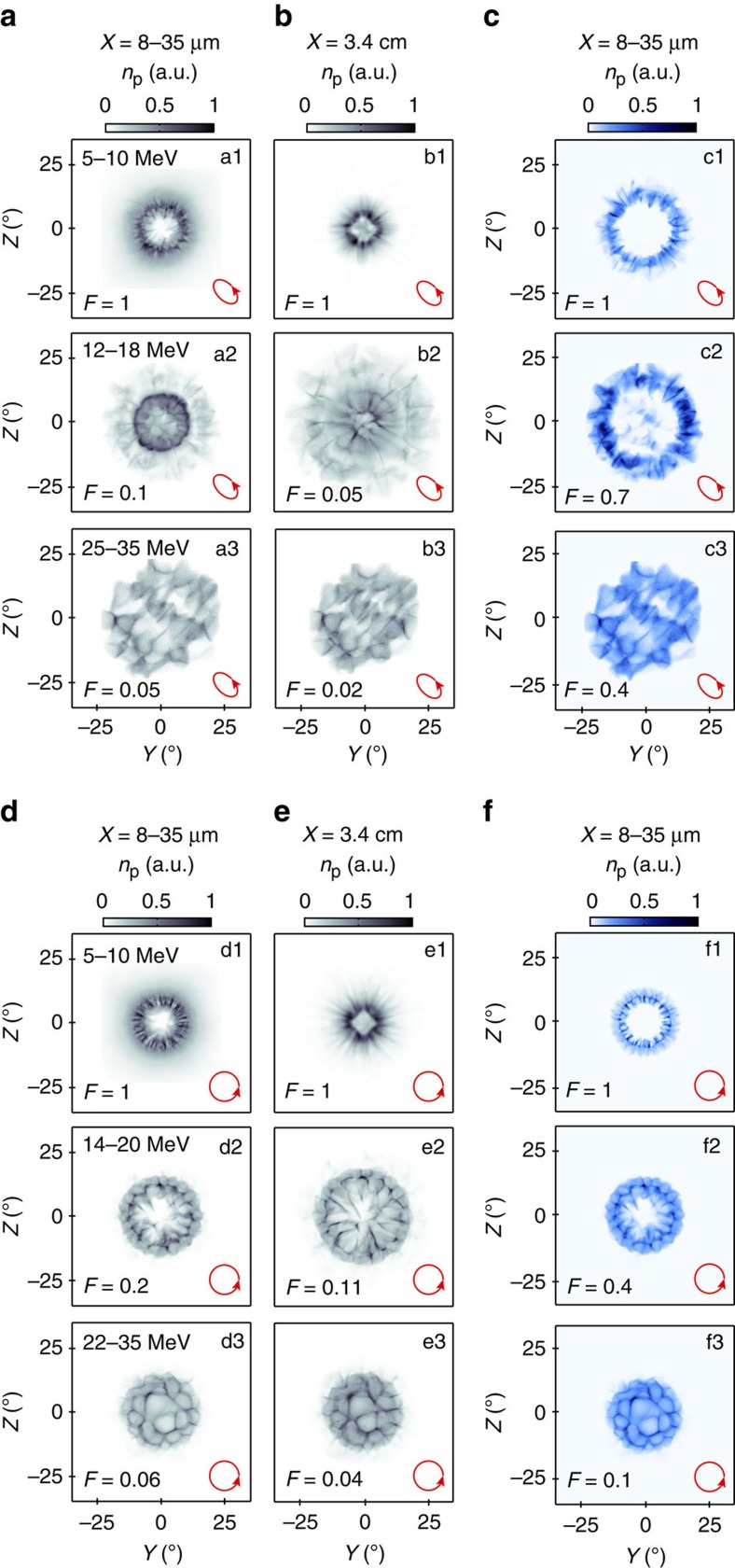
3D-PIC simulations results showing the proton spatial-density distribution for elliptical and circular polarization. (**a**–**c**) Same caption as Fig. 2d–f, for elliptically polarized light. (**d**–**f**) Same for circularly polarized light.

The equivalent simulation results for elliptical polarization, shown in Fig. 3a–c, exhibit similar proton beam profiles to the linear case, but rotated by 45°, as defined by the orientation of the major axis of the polarization ellipse (in the *Y*–*Z* plane). Both the high-density ring at low proton energies and the striped population at higher energies are produced. The increase in beam size between 5 and 18 MeV is very similar to that observed experimentally in Fig. 1f and in both cases a large ring is produced at ∼18 MeV, which is orientated at an angle close to the major axis of the polarization ellipse and has radial modulations.

Finally, the circular polarization case, shown in Fig. 3d–f, also exhibits a small, high-density ring at low energies of 5–10 MeV and a spatially larger, lower-density ring at 14–20 MeV, both showing strong radial features and circular symmetry. This overall change in beam profile is similar to the measurement shown in Fig. 1g. With increasing energy (22–35 MeV), the beam is modulated with bubble-like structures, which are circular and distinctly different from the stripe patterns for linear and elliptical polarization. Similar bubble-like structures are observed experimentally, as shown in the inset of Fig. 1g, and are described by Sgattoni *et al*.^29^ as arising due to a laser-driven Rayleigh–Taylor instability. Compared with the linear and elliptical polarization cases, a larger percentage of the protons produced by circularly polarized light originate at the target front side (as observed in Fig. 3f), which is consistent with previous results indicating more efficient RPA when using circular polarization to reduce electron heating and thereby target expansion^31^.

Thus, in all three polarization cases characteristic features in the measured proton beam, including changes to the beam size and spatial-intensity structure as a function of energy, are observed in the simulation results projected to *X*=3.4 cm. The simulations further show that the individual proton beam distributions are strongly correlated to the collective response of the plasma electrons to the near-field diffraction pattern of the intense laser light transmitted through the self-formed relativistic plasma aperture. The electron beam profile is mapped into the proton beam via modulation of the electrostatic acceleration field, as discussed in the next section.

### Protons sourced at the target front and rear surfaces

**Figure 4:**
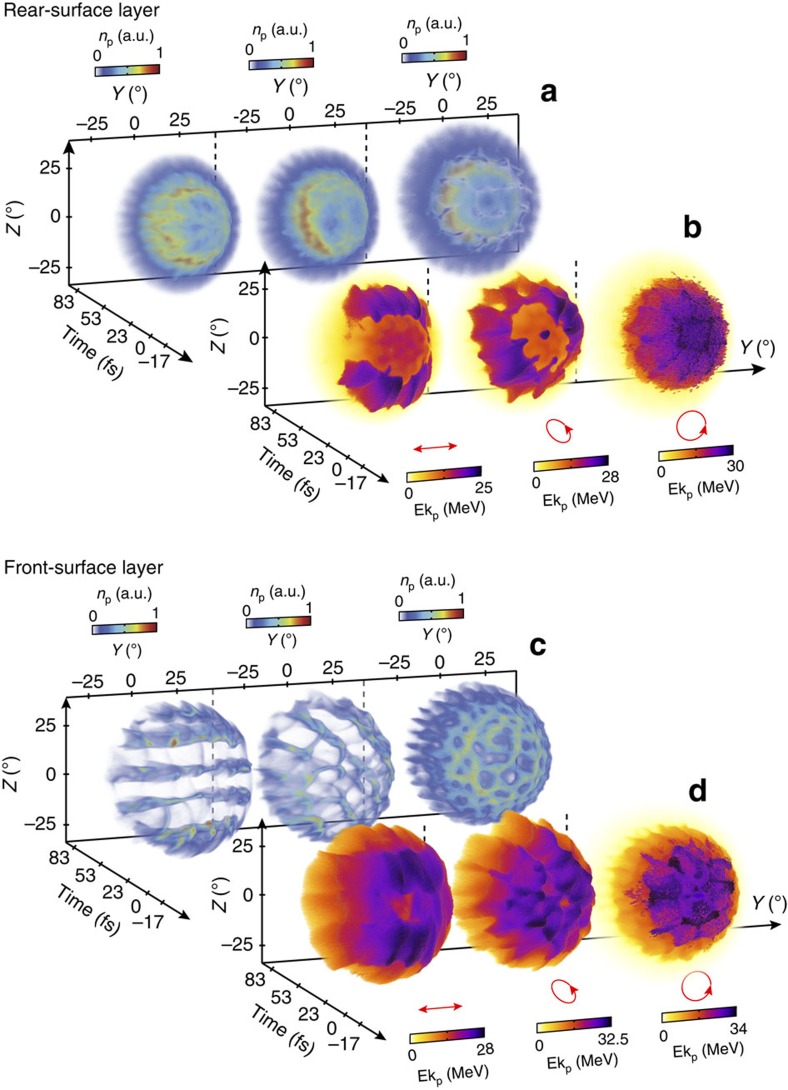
A comparison of spatial-density and kinetic energy distributions as a function of proton origin, with single-source layers. Time-resolved (**a**) spatial-density distribution and (**b**) kinetic energy distribution, at *X*=3 μm, for all three polarization cases (as labelled with the small red arrows) for rear-surface protons. (**c**,**d**) Same, for protons originating at the target front surface.

The results in Figs 2 and 3 clearly show that different structures are produced in the proton populations accelerated at the front and rear sides of the target foil. To examine this aspect further, we have performed separate simulations with a single hydrocarbon layer on either the target front or rear side, for all three polarization cases. The resulting proton density and kinetic energy distributions in the *Y*–*Z* plane as a function of time are shown in Fig. 4.

These additional simulation results show that protons at the target rear form a ring distribution in density and close examination shows that the density at different points around the ring varies with polarization. Importantly, it is clear that in the case of linear and elliptical polarization the highest total proton energies are produced perpendicular to the polarization axis (or the major axis of polarization in the latter case). With circularly polarized light the highest proton energies are observed in a radially modulated pattern within the ring. The proton distribution accelerated from the front-surface layer by contrast is heavily modulated in density, with the striped or bubble-like pattern defined by the polarization, as discussed in the previous section. The proton energy distributions show evidence of the same modulation structures, although the energy is more uniformly distributed about the proton beam front compared with the equivalent rear-surface case.

**Figure 5:**
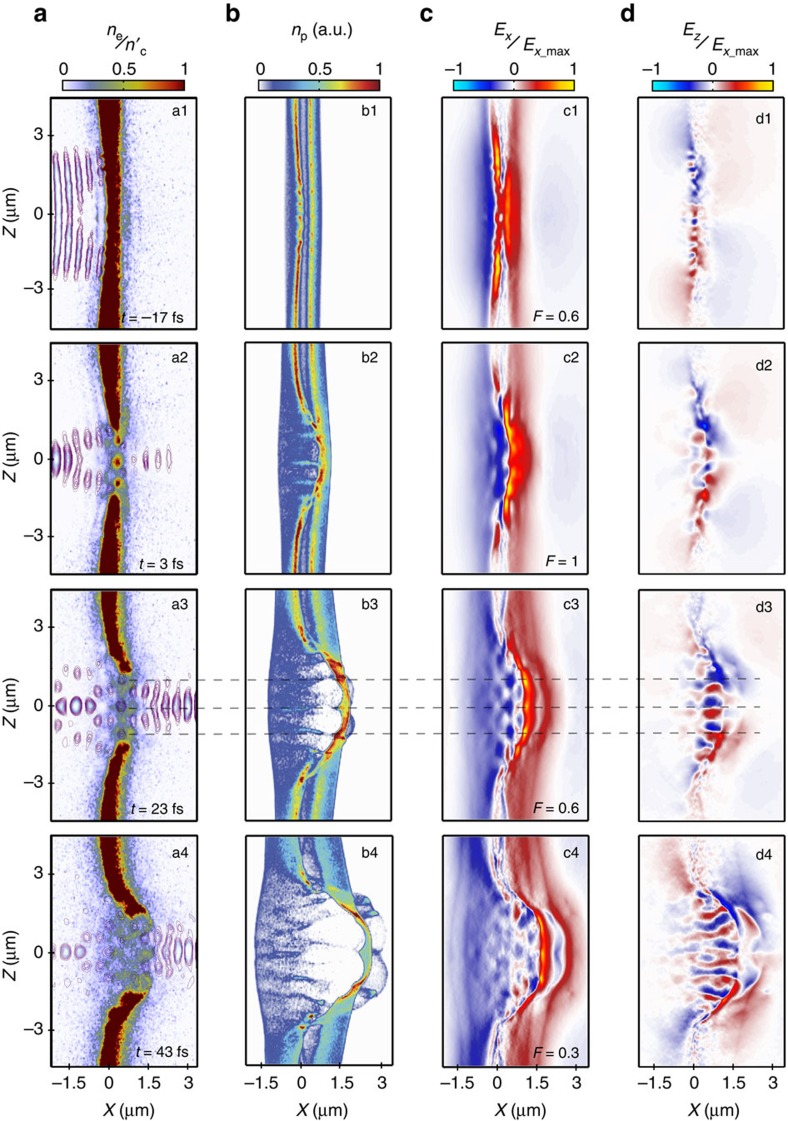
Electrostatic field and particle density evolution with protons on both surfaces. (**a**) Laser field contours and electron density in the *X*–*Z* plane at *Y*=0 at (**a1**) *t*=−17 fs, (**a2**) *t*=3 fs, (**a3**) *t*=23 fs and (**a4**) *t*=43 fs; the onset of transparency and laser diffraction is observed. (**b**) Same for proton density, showing the front-surface protons being accelerated into the rear-surface population. (**c**,**d**) Same for the longitudinal and transverse electrostatic field components respectively, resulting from charge separation, demonstrating the onset of transverse modulations. The simulation time sampled is the same in all four sub-plots. The laser is linearly polarized along the *Y* axis and the target has hydrocarbon layers on both the front and rear surfaces. The dashed lines mark the positions in *Z* of high-density electron lobes and are interleaved with maxima in the near-field laser diffraction pattern. The electrostatic field is strongly modulated at these *Z* positions, with maxima corresponding to regions of high electron density. The beam of accelerated protons thus forms stripes in the *Z* axis.

**Figure 6:**
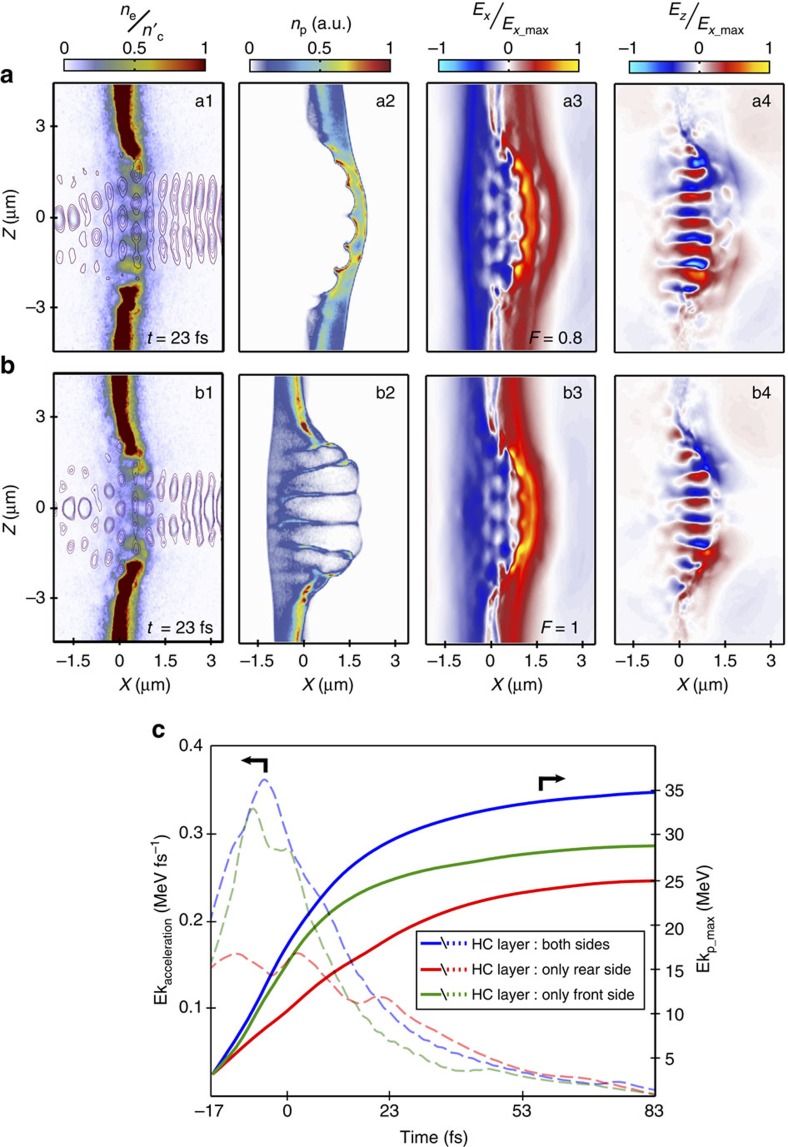
Comparison of electrostatic field and particle density distributions for front-only and rear-only proton sources. (**a**,**b**) From left to right (as in Fig. 5): laser field contours and electron density; proton density; and longitudinal and transverse electrostatic field components, in the *X*–*Z* plane at *Y*=0 for hydrocarbon layer on the target (**a**) rear surface and (**b**) front surface. *t*=23 fs in both cases. (**c**) Evolution of the maximum proton energy (solid lines) and rate of change in kinetic energy (dashed lines) for three cases, corresponding to hydrocarbon layers on both surfaces (blue), only on the rear surface (red) and only on the front surface (green). In all cases the laser is linearly polarized along the *Y* axis.

The fact that the highest-energy protons are sourced at the front surface of the target is consistent with the higher proton energies expected from the RPA mechanism, compared with rear-surface TNSA, for the laser pulse and target parameters considered. We explore the dynamics of the two proton layers further by plotting the laser intensity, electron density, proton density and electrostatic field components in the *X*–*Z* plane at sample time steps in the interaction, before and after transparency has occurred. The results for the case with hydrocarbon layers on both surfaces is presented in Fig. 5. Relativistic transparency occurs at *t*=−5 fs, that is, 5 fs before the peak of the laser reaching the target. The cases for rear-surface-only and front-surface-only are presented in Fig. 6a,b, for which transparency occurs earlier in the interaction, at *t*=−16 and −13 fs, respectively. In all cases the laser is linearly polarized (along the *Y* axis) so that the formation of the striped pattern can be investigated.

The temporal sequence in Fig. 5a shows compression of the target electron layer, formation of the relativistic plasma aperture and modulation of the plasma electron density (Fig. 5a2) due to the near-field diffraction pattern of the intense laser light passing through the aperture. The longitudinal displacement of electrons arising from the transverse ponderomotive forces results in transverse modulations in the resulting longitudinal electrostatic field (Fig. 5c3), which gives rise to the striped proton beam profile (Fig. 5b3). Note that the observed deformation of the target close to the edge of the aperture is produced by radiation pressure, where the laser intensity is high, but not above the threshold for RIT.

The plasma aperture formed in the target with protons on both surfaces is slightly smaller than the cases with protons on only one surface. Transparency also occurs slightly later in the interaction because the effective target thickness is increased by ∼38% by the presence of a second hydrocarbon layer, requiring the plasma electron population to expand further before RIT occurs. The difference in aperture size changes the number of diffraction lobes produced in the region of the highest electron density, as discussed in ref. 27. Whereas two diffraction maxima (regions of intense laser light) are formed in the case with protons on both surfaces, as shown in Fig. 5a, four maxima are formed due to the larger aperture produced with protons on one surface only (either surface), as shown in Fig. 6.

The double-layer simulation reveals that protons from the front side (in the region of the laser focus) are accelerated to higher energies than those from the rear and are driven through the sheath-accelerated rear-surface population, as shown in the temporal sequence in Fig. 5b. An on-axis and two off-axis peaks in the electrostatic field are produced in the *Z* direction (at *Z*=0 and ±1 μm in Fig. 5c3) by the transverse displacement of electrons (Fig. 5a3). These transverse field modulations seed regions of higher proton density in the *Z* direction (Fig. 5b3) when the proton distribution expands transversely to form a ring-like distribution downstream. The three dashed lines in Fig. 5 show how features in the laser near-field diffraction profile map into the electrostatic field components and subsequently into the beam of protons.

The larger aperture produced in the single-layer simulations results in five peaks in the electrostatic field in the *Z* direction. The rear-surface-only simulation shows that the slowest protons at the back of the sheath-accelerated layer are subjected to the field modulations, resulting in the seeding of the density modulations shown in Fig. 6a2. In the front-surface-only simulation the higher-energy protons accelerated by radiation pressure propagate through the target and are strongly influenced by the transverse modulated electrostatic field. The fields in both the positive and negative *X* directions are modulated in the same way and hence the protons passing through this structure are deflected into a striped pattern. Figure 6b shows that the front-surface proton contours coincide with the peaks in the modulated electrostatic field. This radiation-pressure-driven population is also more divergent than the rear-surface TNSA protons (due to the transverse variation in radiation pressure), and the result is the large, striped halo distribution observed in Fig. 2d3, [Fig f3] and measured in Fig. 1e.

It is interesting to note that in the simulation results for all three polarization cases (only the linear case is shown for brevity), the highest-energy protons are produced due to the radiation pressure at the target front side, but that the maximum proton energies are obtained when hydrocarbon layers are present on both surfaces. This is shown in the example results in Fig. 6c, in which the maximum proton energy and the rate of increase in the proton energy are plotted as a function of time. Several conclusions can be drawn from this. First, although the acceleration time is slightly longer for the rear-surface protons, the radiation pressure accelerated front-surface protons reach a higher energy because the magnitude of the acceleration is almost a factor of two higher. Second, the overall higher proton energies in the case of the dual proton layer results from a combination of the high magnitude of acceleration of the front-surface layer and the longer acceleration time due to RIT occurring slightly later in the laser-foil interaction (because of the ∼38% increase in the overall target thickness discussed above).

Finally, we note that a similar analysis performed for a thicker (200 nm) target simulation (not shown), with identical laser pulse parameters and for which transparency does not occur, produced a smaller maximum proton energy (∼10 MeV). In this case the magnitude of acceleration is smaller because the target areal density is higher. The maximum ion energy produced by RPA scales inversely with the areal density^32^.

## Discussion

By using laser polarization to control the collective plasma electron response to the diffraction of intense laser light in ultra-thin foils, we have demonstrated that the spatial-intensity profile of the beam of accelerated protons can be manipulated. Our detailed simulation results indicate that it is possible to use this approach to vary the proton beam profile. The experiment results show that distinctive structures in the proton beam, such as rings, bubbles and striped distributions, are changed by variation of the degree of ellipticity in the laser polarization. The simulation results demonstrate that the electron beam structure is mapped into the proton beam via transverse modulation of the electrostatic field that is produced by charge displacement. The simulation results further show that both the RPA and TNSA ion populations are affected by the modulated field structure and in different ways. Thus, the combination of selective coating of a target foil, with variation of laser polarization may enable manipulation of the collective dynamics of energetic ions produced by either acceleration mechanism. Development of this approach may enable optical control over the shape, divergence and energy profile of beams of laser-accelerated protons. This could enable active proton beam tailoring at the high repetition rates required by many potential applications of intense laser-driven ion sources.

## Methods

### Experiment

The two-dimensional spatial-intensity distribution of the proton beam at selected energies was measured using stacked dosimetry film (radiochromic film) with mylar filters. The detector stack was positioned 3.4 cm downstream from the rear surface of the target. Two types of dosimetry film, HDV-2 and EBT-2, were used to enable the detection and resolution of both low-energy, high-density and high-energy, low-density portions of the proton beam. The higher-sensitivity EBT-2 film was unavailable for the linear polarization shots and hence the high-energy portion of the beam could not be measured for those shots. In all cases an aluminium foil was placed in front of the first radiochromic film layer to remove any potential contribution from the laser light and accelerated carbon and aluminium ions. The experiment was performed using the Gemini Ti:Sapphire laser at the Rutherford Appleton Laboratory in the United Kingdom^33^. The laser was optimized to work at a central wavelength of 800 nm and delivered pulses with duration equal to 40 fs, FWHM. A double plasma mirror configuration was used to enhance the intensity contrast to ∼10^11^ and ∼10^8^, at 1 ns and 2 ps, respectively, before the peak of the pulse. The final energy in the laser pulse was (2.0±0.2) J and it was focused, using an off-axis *f*/2 parabola, along target normal onto the front surface of a 10 nm-thick aluminium foil target, to a focal spot diameter of 3 μm (FWHM). The calculated peak intensity is 6 × 10^20^ W cm^−2^. A deformable mirror was used before the focusing parabola to ensure a high-quality focal spot on target. The laser beam was switched between linear (Δ*θ*=0), elliptical (Δ*θ*=*π*/4) and circular (Δ*θ*=*π*/2) polarization using thin mica wave plates (where Δ*θ* is the phase difference between the two orthogonal components of the laser beam).

### Simulations

The simulations were performed using the fully relativistic, 3D PIC code, EPOCH^34^. Each simulation was defined with Cartesian spatial dimensions of 20 μm × 20 μm × 20 μm using 1,000 × 720 × 720 computational mesh cells. An 800 nm-wavelength laser pulse was injected into this computational domain with a Gaussian temporal profile of 40 fs (FWHM), focused to a Gaussian spatial intensity profile of 3 μm FWHM. The pulse had a peak intensity of 6 × 10^20^ W cm^−2^. Thus, the pulse parameters were chosen to match those of the experiment. For each simulation, the polarization of the laser was selected to be either linear, elliptical (ellipticity of *π*/4) or circular. The target in each simulation was a representation of a solid density 10 nm-thick (Al^13+^) slab with a 6 nm-thick C^6+^ and H^+^ hydrocarbon contamination layer (of composition C_2_H_6_) defined on either the front, rear or both surfaces. To have an adequate number of computational cells across the target, both the target slab and the contamination layer(s) were pre-expanded with a one-dimensional 245 nm FWHM Gaussian profile. This reduces the peak electron density to 14.3*n*_c_ and 3.7*n*_c_ for the main target and contamination layer(s), respectively. The ion densities are set to neutralize the electron charge appropriately and the initial electron temperature was defined as 100 keV. This temperature and expansion is justified as experimentally the target expands due to electron heating driven by the rising edge of the laser pulse intensity profile. The boundaries of the simulation box are all defined as free space. The laser enters from the left boundary.

### Data availability

Data associated with research published in this paper can be accessed at http://dx.doi.org/10.15129/cb8de272-7651-4ac8-8eea-3961291e1e30.

## Additional information

**How to cite this article:** Gonzalez-Izquierdo, B. *et al*. Towards optical polarization control of laser-driven proton acceleration in foils undergoing relativistic transparency. *Nat. Commun.* **7**:12891 doi: 10.1038/ncomms12891 (2016).

## Supplementary Material

Supplementary Information Supplementary Figures 1-9 and Supplementary Discussion.
