# Supplementary material for: Towards optical polarization control of laser-driven proton acceleration in foils undergoing relativistic transparency
Source: Nat Commun. 2016 Sep 14;7:12891. doi: 10.1038/ncomms12891 (PMC5027290; doi:10.1038/ncomms12891)
Supplement: Supplementary Information — Supplementary Figures 1-9 and Supplementary Discussion. [file ncomms12891-s1.pdf]

Supplementary Figures

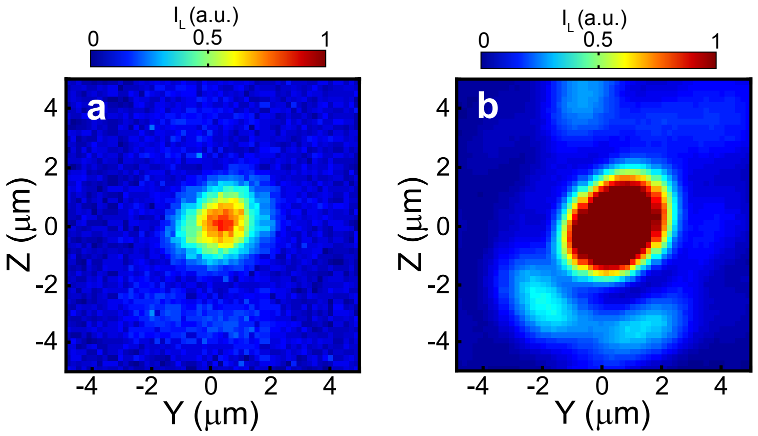

Supplementary Figure 1: **Measured laser focal spot for the experiment reported in the main paper.** **a**, Laser spatial-intensity profile at best focus (measured at low laser energy to prevent damage to the camera and optics). **b**, Same as **a** but with the image saturated in the centre to show the 'shape' of the focus.

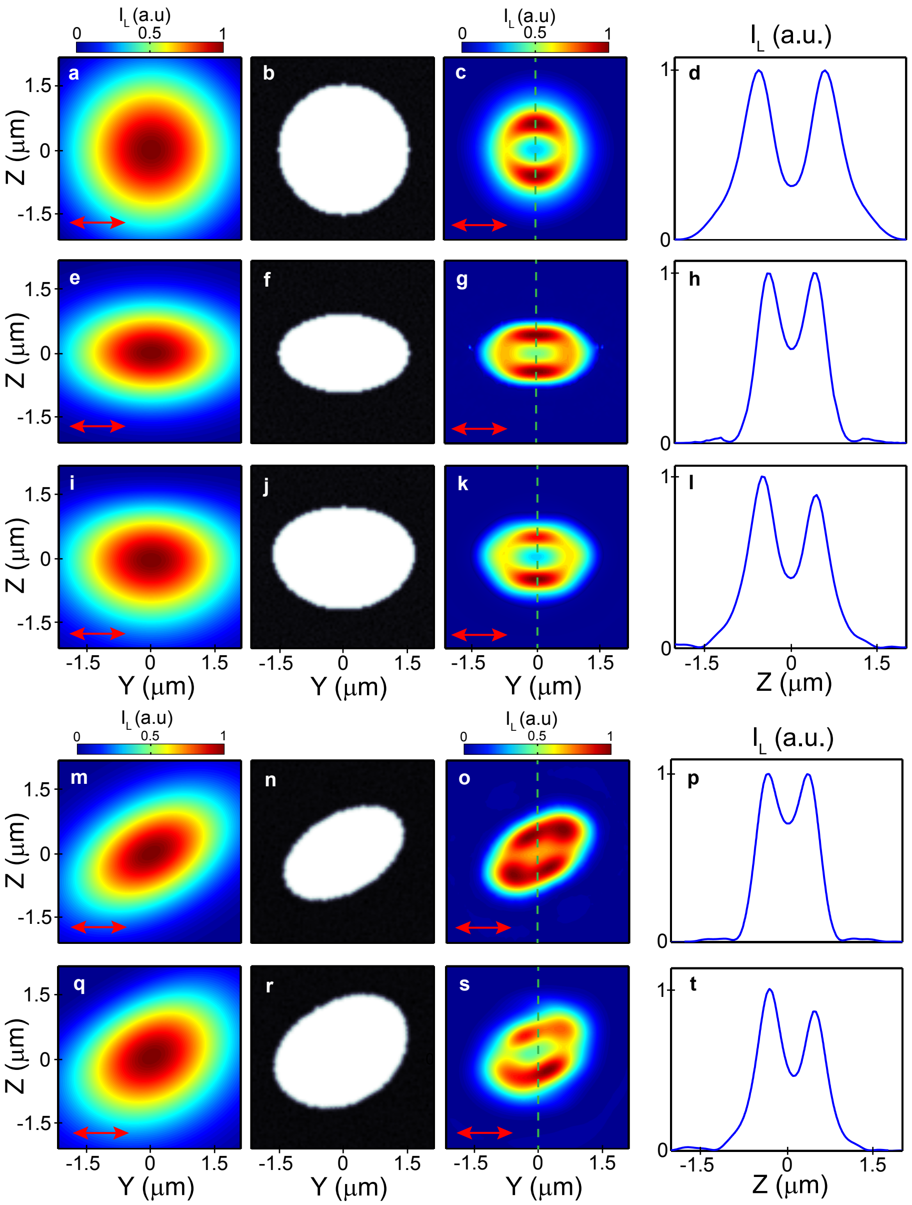

Supplementary Figure 2: Intensity diffraction pattern produced by a fixed aperture, as defined by the laser focal spot shape. **a**, The laser focal spot intensity distribution for a circular Gaussian distribution. **b**, A fixed plasma aperture as defined by an iso-intensity contour within the laser focal spot in **a**. **c**, The resulting near-field diffraction pattern in the transmitted laser light, exhibiting the familar double-lobe intensity structure. **d**, A lineout along the intensity distribution in **c** (green dashed line), which demonstrates that a circular focal spot produces a uniform diffraction pattern with two vertical lobes. The subsequent rows of panels exhibit the corresponding data for different focal spot distributions: **e-h** an ellipse which is symmetrical in the Z-Y axes, **i-l** an ellipse with symmetry only in the Y axis, **m-p**, a symmetrical ellipse rotated by 30° **q-t**, an assymetrical ellipse which is then rotated by 30°.

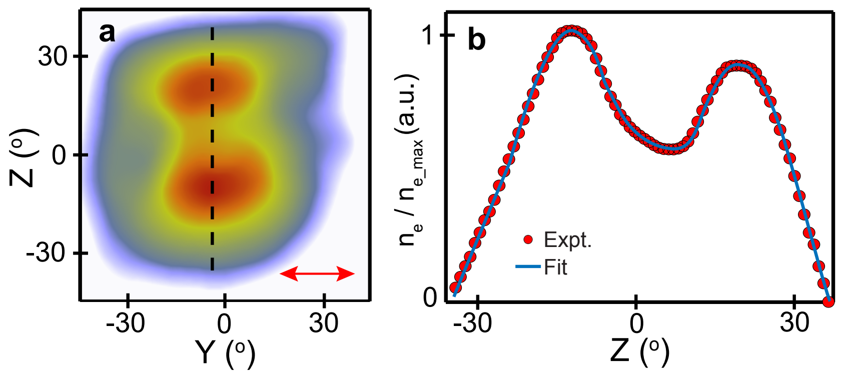

Supplementary Figure 3: Experimental results showing measurements of the electron density distribution, a, Electron density as measured using image plate for electrons greater than 3.5 MeV **b**, Integrated signal along the dashed line in **a**.

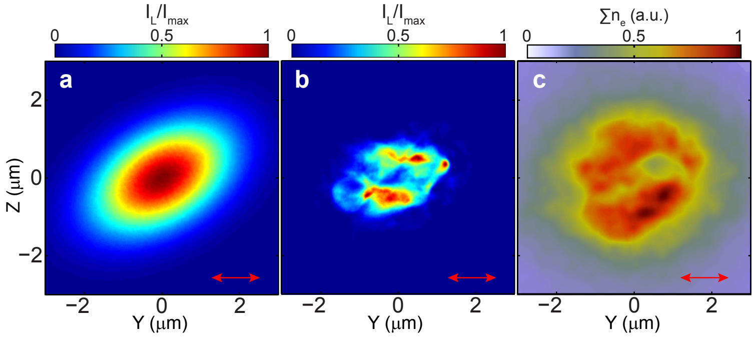

Supplementary Figure 4: Full intensity 3D PIC simulation results with an elliptical focal spot distribution. a, Elliptical laser focal spot in the plane of the target, rotated by 30° and normalised to the maximum laser intensity. **b**, Laser focal spot after diffracting through the self-induced relativistic plasma aperture. **c**, The resulting electron density distribution driven by the transverse ponderomotive forces within diffracted laser intensity profile.

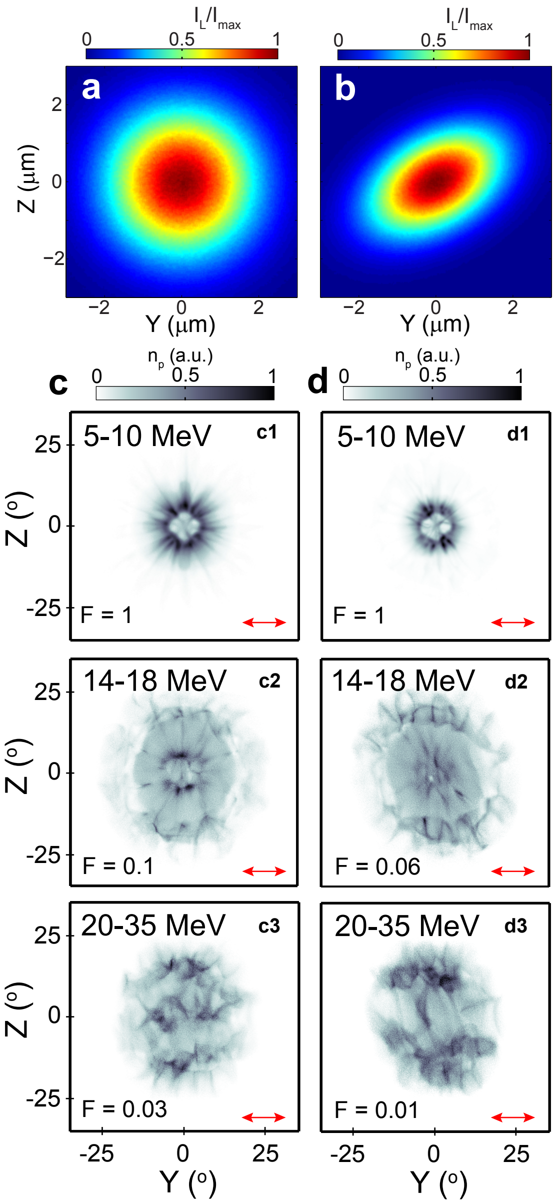

Supplementary Figure 5: **Comparison of proton beam density profiles from 3D PIC simulations using circular and elliptical laser focal spots. c1-c3,** Proton density distribution for given ranges of proton energies, as driven by the circular laser intensity profile shown in **a**. **d1-d3**, Same as **c** but driven by the elliptical, rotated focal spot shown in **b**.

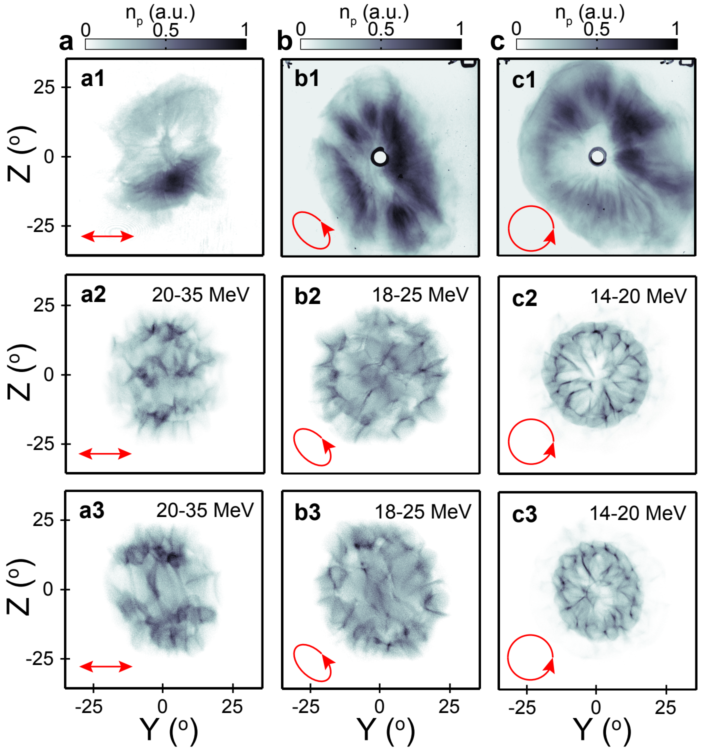

Supplementary Figure 6: Comparison of experiment and 3D PIC simulations of the proton beam spatial-intensity profile. a1, Measured proton spatial intensity profile of MeV protons driven by a linearly polarized laser pulse. The corresponding measured laser focal spot is shown in Supplementary Fig. 1. **a2**, Proton beam spatial-intensity profile from 3D PIC simulations using a circular laser focal spot, as shown in Supplementary Fig. 5a. **a3**, Proton beam spatial-intensity profile from 3D PIC simulations using a rotated elliptical focal spot, as shown in Supplementary Fig. 5b. **b1-b3**, Same for an elliptically polarized laser pulse. **c1-c3** Same for a circularly polarized laser pulse.

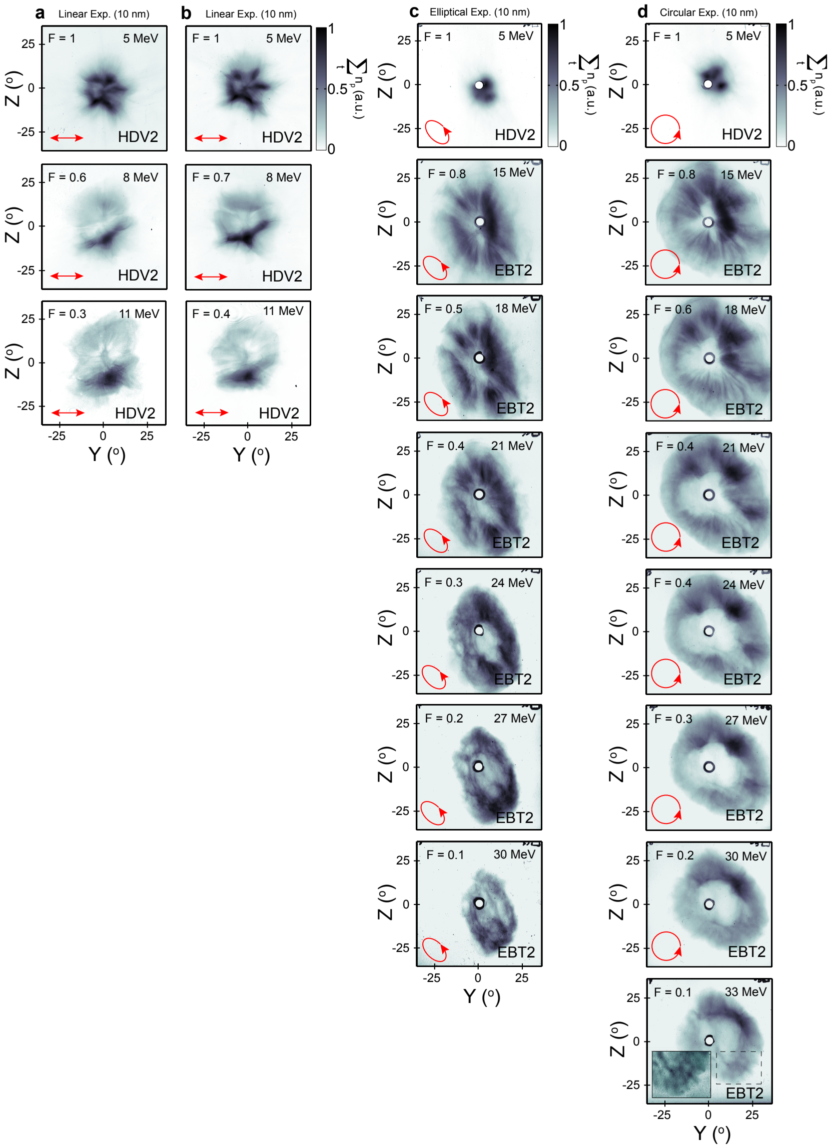

Supplementary Figure 7: Additional measured proton spatial-intensity profiles from the experiment. **a-b**, Measured proton beam spatial-intensity profiles as a function of energy for two separate laser shots with peak intensity within 5%. The target thickness is 10 nm and the laser is linearly polarized in both cases. Key features including the asymmetrical and tilted double lobe distribution are fully reproduced. **c**, Same for elliptically polarized laser light. **d**, Same for circularly polarized light.

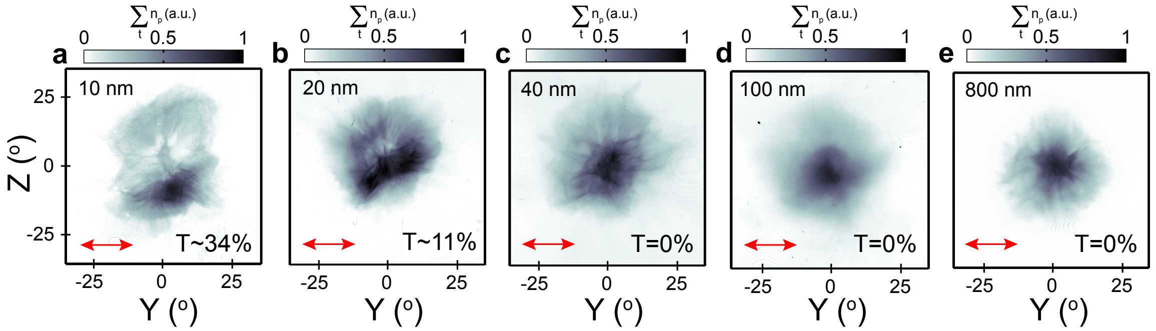

Supplementary Figure 8: **Measured proton spatial-intensity profiles (at 11 MeV) as a function of target thickness for linearly polarized light. a,** 10 nm Al; **b**, 20 nm Al; **c**, 40 nm Al; **d**, 100 nm Al; **e**, 800 nm Al. The transmitted light fraction is included for each target thickness case.

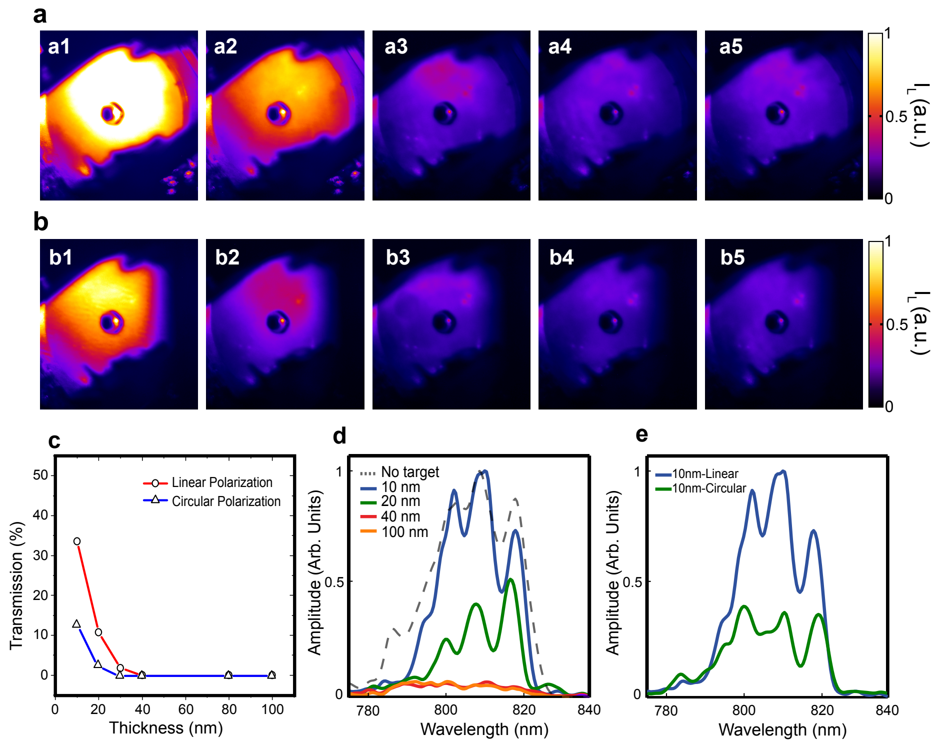

Supplementary Figure 9: **Measurements of the laser light transmitted through the target. a,** Images of the spatial-intensity profile of the laser light transmitted through the target, as a function of target thickness: **a1**, 10 nm; **a2**, 20 nm; **a3**, 40 nm; **a4**, 80 nm; **a5**, 100 nm; for linear polarization. **b**, Same for circular polarization. **c**, Integrated measurements of the transmitted laser energy (from the results in **a** and **b**) normalized to the laser pulse energy, as a function of target thickness. **d**, Measurements of the spectrum of the transmitted laser light for a range of target thicknesses. **e**, Measurements of the spectrum of the transmitted laser light for 10 nm Al for linear and circular polarization.

Supplementary Discussion

I. INTRODUCTION

In this Supplementary Information file, additional experimental and simulation results are presented. In Section II, an investigation of the influence of the shape and intensity distribution of the laser focus on the near-field diffraction pattern of the transmitted laser light and subsequently on the electron and ion spatial intensity profiles is presented. This includes the measured laser focal spot distribution and 3D PIC simulations using this distribution. In Section III, additional experimental data showing the spatial-intensity distribution of the proton beam as a function of energy, for given polarizations, and as a function of target thickness, is presented. Finally, in Section IV, experimental results quantifying the percentage of laser light transmitted through the target and the spectrum of the light, as a function of target thickness and polarization, are presented.

II. INFLUENCE OF THE LASER FOCAL SPOT DISTRIBUTION

Although the simulations presented in the main paper all involve a circularly symmetric focal spot, we note that in a typical high power laser experiment the actual spot achieved will deviate from this ideal scenario. In this section, we consider the extent to which this deviation would affect the resulting proton beam profile. We begin by considering the actual focal spot measured in the experiment, which is shown in Supplementary Figure 1. Although this is considered to be a good quality focal spot, a detailed analysis shows that it is slightly elliptical (with a major-to-minor axis ratio of 5:3) and with the major axis at an angle of $\sim 40^{\circ}$ with respect to the linear polarization axis (Y-axis in Supplementary Figure 2). We have performed a series of simulations to investigate what effect this focal spot shape has on the final proton beam spatial-intensity profile.

We begin by considering the influence of the focal spot shape and orientation on the laser near-field diffraction pattern. We base this investigation on linearly polarized light, with the polarization in the Y-axis, as in the main paper. The first simulations are performed for a simplified case with a fixed, pre-defined aperture (i.e. without plasma). In this case the aperture shape is defined by the laser focal spot shape, i.e. the profile of the

threshold iso-intensity condition above which relativistic induced transparency occurs (i.e. $n_e < n_c'$ where n_e is the plasma electron density, $n_c' = \gamma m_e \epsilon_0 \omega_L^2 / e^2$ is the relativistically corrected critical plasma density, γ is the electron Lorentz factor, m_e is the electron rest mass and ϵ_0 is the vacuum permittivity). Supplementary Figure 2 shows how the laser diffraction pattern varies when the focal spot deviates from circular symmetry. For a circularly symmetric focal spot the diffraction through the fixed aperture produces two lobes, one either side of the laser polarization axis. In this case there is a uniform distribution of laser intensity into the two lobes, as shown by the vertical line out in Supplementary Figure 2d. We now investigate the effect of transforming the circular focal spot into a symmetric elliptical focal spot, in Supplementary Figure 2e-h. Two symmetric diffraction lobes are still produced along the same axis, but their separation is smaller, and defined by the size of the aperture along the Z-axis. In Supplementary Figure 2i-l, the effect of inducing a slight asymmetry to the laser focal spot is investigated. Whilst the near-field diffraction pattern still contains two lobes, the intensity distribution in the lobes is no longer the same. Thus, it is shown that the overall transmitted laser beam profile continues to be dominated by near-field diffraction, but that the separation and symmetry of the diffraction lobes is influenced by changes to the shape and symmetry of the laser focal spot, respectively.

Next we consider the effect of rotating the laser focal spot with respect to the polarization axis, as is the case for the measured focal spot distribution in Supplementary Figure 1. Supplementary Figures 2m-p and q-t show the effect of rotating the focal spot for a symmetrical ellipse and asymmetrical focal spot, respectively. The double diffraction lobe distribution is still produced, one lobe either side of the polarization axis, but the overall distribution is distorted such that it follows the general shape of the laser focus. Note that this is not simply a rotation of the double-lobe distribution - the minimum axis between the lobes remains along the laser polarization axis, but the distribution of the laser light intensity within the lobes has changed.

The near-field diffraction pattern of the transmitted laser light (within 1-2 μm of the relativistic plasma aperture) cannot be measured experimentally. However, Supplementary Figure 3 shows measurements of the spatial-intensity distribution of the beam of electrons displaced by the transverse ponderomotive force produced by the laser near-field diffraction pattern. The electron intensity distribution exhibits a double lobe con-

figuration slightly rotated with respect to the laser polarization axis, as shown in Supplementary Figure 3a. Additionally, there is an intensity-asymmetry between the two lobes, as highlighted in Supplementary Figure 3b. Given that the measured laser focal spot is in fact a rotated, slightly asymmetric ellipse, the measured asymmetry in the electron density distribution between the two lobes is consistent with the expected diffracted laser intensity profile shown in Supplementary Figure 2. As will be shown below, this asymmetry and rotation is mapped into the proton beam profile.

The modelling discussed thus far considers the effects of the laser focal spot distribution on the near-field diffraction pattern after propagation through a fixed plasma aperture, i.e. a simplified case without inclusion of laser-plasma interaction effects. We next perform a 3D simulation with a rotated elliptical focal spot with peak laser intensity equal to 6×10^{20} Wcm^{-2}, which is comparable to the experiment and with which we investigate the relativistic plasma dynamics. With the exception of the laser focal spot distribution, the simulation parameters are the same as those stated in the Methods section of the main paper.

Supplementary Figure 4a shows the input focal spot distribution, which is a rotated ellipse with similar parameters to the measured experimental focal spot distribution, and Supplementary Figure 4b and c show the near field diffraction profile in the transmitted laser beam and in the resulting electron distribution, respectively. The asymmetric double-lobe distribution in Supplementary Figure 4b is in very good agreement with the expected distribution, as modelling in Supplementary Figure 2s. The electron distribution in Supplementary Figure 4c is qualitatively very similar, in terms of distortion to the double lobe structure, to the measured electron beam profile in Supplementary Figure 3a.

Importantly, both the simplified, predefined aperture simulations and the full intensity laser-plasma simulations show that the transmitted laser beam profile close to the target is dominated by near-field diffraction effects. The spatial-intensity profile of the laser spot is shown to influence the laser intensity distribution within the near-field diffraction pattern, but not to fundamentally change the beam profile.

From the same simulations the proton spatial-intensity profile as a function of energy was also extracted, and is shown in Supplementary Figure 5. The profile is extracted late in the simulation, at a time much greater than the laser pulse duration, at which the

proton dynamics have reached a quasi-steady state. In order to investigate the energy dependence of the measured proton spatial-intensity distribution, and to relate back to the experimentally measured energy-resolved distribution, a series of integrations in specified energy bands are made. In Supplementary Figure 5c1-c3 proton profiles are shown for an idealised circular focal spot (shown for clarity in Supplementary Figure 5a). These results, which are presented and discussed in detail in the main paper, exhibit a lobed distribution about the axis of polarization, particularly at high energies. Importantly, in the case of the rotated, elliptical focal spot, we find that the overall proton beam distribution exhibits the same overall lobed features, but that the distribution is distorted slightly along the Y-axis, to give the appearance of a slight angular rotation, as shown in Fig. 5d1-d3. We note that this distribution is in closer overall agreement to our measured proton beam distribution, as shown in Supplementary Figure 6a.

Having established the effect on linear polarization, in Supplementary Figure 6 corresponding results for the effect on the elliptical and circular polarization cases are also presented. For all three polarization cases, it is found that the proton beam is slightly compressed along the major axis of the laser ellipse, and as a result give better overall agreement to the proton beams measured in the experiment (also shown). The overall features are however set by the near-field diffraction pattern of the transmitted laser light.

We conclude from this study that an asymmetry in the laser focal spot intensity distribution will induce asymmetry in the diffraction pattern, which maps into the transverse expulsion of electrons and thus into the resulting electrostatic field and ultimately the proton beam. However, the overall beam profile is set by the near-field diffraction pattern of the transmitted laser light and thus mainly defined by the polarization of the light.

III. ADDITIONAL EXPERIMENTAL RESULTS

In Supplementary Figure 7, the full energy-resolved spatial-intensity distribution measurements of the proton beam are presented for all three polarization cases. Supplementary Figure 7a corresponds to the linear polarization case included in the main text. In addition, results from a second shot with linear polarization are presented in

Supplementary Figure 7b, which clearly shows the reproducibility of the results. Both the asymmetrical beam density in the lobes and the tilt with respect to the polarization axis, which are produced by the focal spot distribution, as discussed above, are clearly observed in these results.

Note that the difference in laser pulse intensity for the two sets of data for linear polarization is <5%. For larger differences in intensity the beam patterns are observed to change. This is because the relativistic plasma aperture size and resulting laser diffraction profile depends on the peak laser intensity. The results reported here were only obtained for laser shots within a narrow range of the maximum intensity achievable using the Gemini laser. Shot-to-shot fluctuations, coupled with the extremely delicate nature of the 10 nm-thick Al targets, which required dedicated vacuum chamber pump-downs for each shot, limited the number of shots contributing to the overall experimental data set.

In Supplementary Figure 8, additional measured proton beam profiles (for linearly polarized light at a single proton beam energy slice) are presented as a function of target thickness. For the 800 and 100 nm cases the beam density peaks in the centre, as expected for TNSA. At 40 nm the target is still opaque to the laser light, as will be demonstrated in the next section, and for targets thinner than this the proton beam splits to form the double lobe distribution. The 10 nm case has been discussed in some detail above. Importantly, we note that the measurement at 20 nm is also fully explained by our model. A double lobe is produced, with an asymmetric distribution (high density in the bottom lobe), and the lobes are tilted at the same angle as the 10 nm case. This is expected because the focal spot distribution is similar in both cases (slightly elliptical and tilted as shown in Supplementary Figure 1). The observation that the lobes are closer together is explained by the fact that the relativistic plasma aperture is smaller in the thicker target - transparency occurs later in the interaction, as discussed in the next section. A smaller aperture results in more closely spaced peaks, as demonstrated in Supplementary Figure 2e-h.

The additional results thus fully support the underlying physics model presented in the paper.

IV. MEASUREMENTS OF TRANSMITTED LASER LIGHT

In Supplementary Figure 9, measurements of the spatial-intensity distribution of the transmitted laser light as a function of target thickness are presented for linear (Supplementary Figure 9a) and circular (Supplementary Figure 9b) polarization. The measurements are made by imaging the laser light transmitted through the target on a PTFE screen positioned 30 cm behind it. The light was collected by a CCD camera with a telephoto lens and wavelength filtered for 800 nm using a bandpass filter. Similarly, a fiber optic was positioned to collect a small fraction of the transmitted light and direct it to an optical spectrometer. The transmission is quantified in Supplementary Figure 9c. It is clear from these measurements that transparency occurs in Al targets thinner than 40 nm. The degree of transparency increases with decreasing target thickness because the target decompresses more quickly, such that the peak electron density decreases below the relativistically corrected critical plasma density earlier in the interaction. We further note that a larger degree of transmission is obtained in the case of linearly polarized light, compared to the corresponding thickness with circular polarization. This results from the larger degree of heating of plasma electrons induced by linear polarization.

These observations of changes to transparency based on the overall beam profile are reinforced by measurements of the transmitted laser spectrum. In Supplementary Figure 9d the transmitted laser energy, as determined from the integrated signal, is also observed to decrease with increasing target thickness. Observed spectral modulations are inherent to the laser pulse, although appear to be enhanced by transmission through the target. In Supplementary Figure 9e example transmitted spectra are shown for 10 nm Al for both linear and circular polarization, which again demonstrates larger transmission for linear polarization.
